# All-Fiber Micro-Ring Resonator Based p-Si/n-ITO Heterojunction Electro-Optic Modulator

**DOI:** 10.3390/ma18020307

**Published:** 2025-01-11

**Authors:** Yihan Zhu, Ziqian Wang, Xing Chen, Honghai Zhu, Lizhuo Zhou, Yujie Zhou, Yi Liu, Yule Zhang, Xilin Tian, Shuo Sun, Jianqing Li, Ke Jiang, Han Zhang, Huide Wang

**Affiliations:** 1 State Key Laboratory of Radio Frequency Heterogeneous Integration, International Collaborative Laboratory of 2D Materials for Optoelectronics Science and Technology, Institute for Advanced Study in Nuclear Energy & Safety, Interdisciplinary Center of High Magnetic Field Physics of Shenzhen University, College of Physics and Optoelectronic Engineering, Shenzhen University, Shenzhen 518060, China; zhuyihan2023@email.szu.edu.cn (Y.Z.); wangziqian@szu.edu.cn (Z.W.); 2400233032@mails.szu.edu.cn (H.Z.); 2400233047@mails.szu.edu.cn (L.Z.); 2410232028@mails.szu.edu.cn (Y.Z.); 2060493022@email.szu.edu.cn (Y.L.); 2060493009@email.szu.edu.cn (Y.Z.); tianxilin2023@email.szu.edu.cn (X.T.); 2350453008@email.szu.edu.cn (S.S.); hzhang@szu.edu.cn (H.Z.); 2 School of Computer Science and Engineering, Macau University of Science and Technology, Macau 999077, China; jqli@must.edu.mo; 3 School of Electronic Engineering, Chengdu Technological University, Chengdu 611730, China; chenshin@cdtu.edu.cn; 4 State Key Laboratory of Luminescence and Applications, Changchun Institute of Optics, Fine Mechanics and Physics, Chinese Academy of Sciences, Changchun 130033, China; jiangke@ciomp.ac.cn; 5 Key Laboratory of Organosilicon Chemistry and Material Technology, College of Material Chemistry and Chemical Engineering, Ministry of Education, Hangzhou Normal University, Hangzhou 311121, China

**Keywords:** electro-optic modulator, optical chip, ITO, all-fiber micro-ring resonator, heterojunction

## Abstract

With the rapid advancement of information technology, the data demands in transmission rates, processing speed, and storage capacity have been increasing significantly. However, silicon electro-optic modulators, characterized by their weak electro-optic effect, struggle to balance modulation efficiency and bandwidth. To overcome this limitation, we propose an electro-optic modulator based on an all-fiber micro-ring resonator and a p-Si/n-ITO heterojunction, achieving high modulation efficiency and large bandwidth. ITO is introduced in this design, which exhibits an ε-near-zero (ENZ) effect in the communication band. The real and imaginary parts of the refractive index of ITO undergo significant changes in response to variations in carrier concentration induced by the reverse bias voltage, thereby enabling efficient electro-optic modulation. Additionally, the design of the all-fiber micro-ring eliminates coupling losses associated with spatial optical-waveguide coupling, thereby resolving the high insertion loss of silicon waveguide modulators and the challenges of integrating MZI modulation structures. The results demonstrate that this modulator can achieve significant phase shifts at low voltages, with a modulation efficiency of up to 3.08 nm/V and a bandwidth reaching 82.04 GHz, indicating its potential for high-speed optical chip applications.

## 1. Introduction

With the advancement of information technology, functional devices and systems, including data centers and artificial intelligence systems, require higher data transmission rates, faster data processing speeds, and larger data storage capacities [1]. It has driven the progress of optoelectronic devices and chips, particularly the core component, the electro-optic modulator (EOM) chip, used for loading high-speed electrical signals onto optical signals [2,3,4]. EOMs are required to possess characteristics such as a large modulation bandwidth, high modulation efficiency, and good integration [5,6]. 

Silicon-based electro-optic modulators (EOMs) use the plasma dispersion effect to achieve signal modulation, which entails that variations in the concentration of free carriers within the silicon waveguide lead to alterations in the waveguide’s real and imaginary parts of the refractive index. This phenomenon is harnessed to achieve phase and intensity modulation. Practical structures that have been developed include the carrier-injection type (p-i-n), the carrier-depletion type (p-n), and the capacitive type (MOS) [7]. However, silicon’s intrinsic electro-refractive effect is relatively weak, leading to low modulation efficiency; typical *V*_πL_ for a carrier-depletion modulator is 1.5–2.5 V cm [8]. Consequently, this necessitates the application of substantial voltage swings of several volts or the implementation of relatively long device lengths in the order of millimeters. This, in turn, creates an upper limit of approximately 50 GHz for the bandwidth [9]. In recent years, there has been extensive research on modulators that integrate silicon waveguides with emerging materials, such as graphene [10,11,12], thin-film lithium niobate (TF-LN) [13], GeSn modulators [14], indium phosphide (InP) [15], electro-optic (EO) polymers [16], and transparent conductive oxides including indium tin oxide (ITO) and aluminum-doped zinc oxide (AZO) [17,18], among other novel materials. 

Indium tin oxide (ITO) exhibits significant advantages, including a broad tunable range of carrier concentrations from 10^19^–10^21^ cm^−3^. Its real part of the dielectric constant approaches zero within the communication band, a phenomenon referred to as the epsilon-near-zero (ENZ) effect [19,20]. This ENZ effect endows ITO with a multitude of unique optical characteristics, such as enhanced light–material interactions, substantial nonlinear responses, and strong coupling phenomena. These properties position ITO as a promising candidate for applications in advanced photonic devices, particularly in the realm of electro-optic modulators where such characteristics can lead to improved performance and functionality [21]. Utilizing indium tin oxide (ITO) for effective electro-optic modulation requires only micrometer-scale device lengths, which correspond to bandwidths in the hundreds of gigahertz. The modulation structures of electro-optic modulators often employ either Mach–Zehnder interferometers (MZIs) [22] or micro-ring resonators, with the latter structure exhibiting low power consumption, high integrability, and enhanced light–material interaction [23,24,25].

Here, we present an electro-optic modulator based on an all-fiber micro-ring resonator and a p-Si/n-ITO heterojunction, achieving high modulation efficiency and large bandwidth. This design incorporates ITO, which exhibits an ENZ effect in the communication band. The real and imaginary parts of the refractive index undergo significant changes in response to variations in carrier concentration induced by small bias voltages, thereby enabling efficient electro-optic modulation and overcoming the limitations imposed by the weak electro-optic effect of silicon. The introduced all-fiber micro-ring resonator eliminates the need for spatial optical-waveguide coupling processes, thereby avoiding coupling losses and addressing the high insertion loss of silicon waveguide modulators and the challenges of integrating MZI modulation structures, which typically exhibit high power consumption. Our proposed p-Si/n-ITO heterojunction electro-optic modulator based on the novel all-fiber micro-ring resonator is expected to meet the demands for high-speed optical chips in terms of modulation efficiency and speed.

## 2. Material and Methods

The complex permittivity of indium tin oxide (ITO) adheres to the Drude model [26], which is described as follows:

$$\varepsilon =\varepsilon_{\infty }-\frac{\omega_{p}^{2}}{\omega^{2}-i\gamma \omega }=\varepsilon_{\infty }-\frac{\omega_{p}^{2}}{\omega^{2}+\gamma^{2}}-i\frac{\omega_{p}^{2}\gamma }{\omega {(\omega }^{2}+\gamma^{2})}\;$$

Herein, $\varepsilon_{\infty }$ represents the high-frequency dielectric constant, and ω denotes the angular frequency. The plasma frequency $\omega_{p}$ and the damping factor γ are defined as follows [27]:

$$\omega_{p}^{2}=\frac{Ne^{2}}{\varepsilon_{0}m^{*}}$$

$$\gamma =\frac{e}{\mu_{n}m^{*}}$$

**Figure 1:**
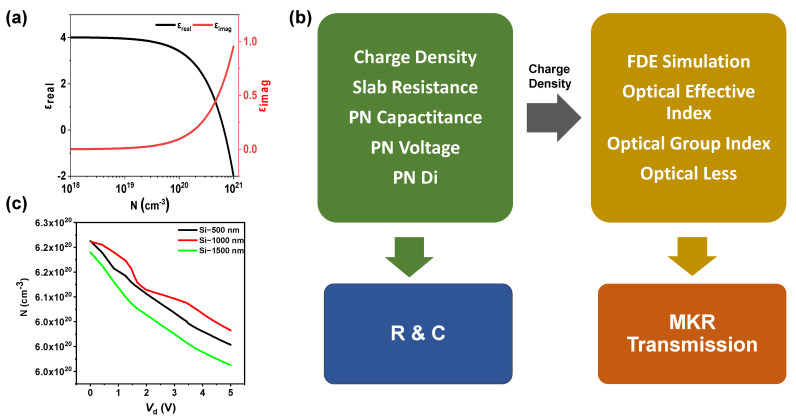
(**a**) Free electron concentration dependence of the real (ε_real_) and imaginary (ε_imag_) parts of the complex permittivity of ITO. (**b**) Complete simulation flow of the proposed p-Si /n-ITO junction electro-optic modulator based on all-fiber micro-ring resonator. (**c**) Voltage-dependent carrier concentration of ITO region in heterojunction devices with different thickness of Si layer.

The carrier concentration N, ranging from 10^19^ to 10^21^ cm^−3^, can be altered by applying an electric field or through the process of doping with varying concentrations of Sn [28,29]. The electron mobility $\mu_{n}=26\;{cm}^{2}V^{-1}s^{-1}$ [30] and the effective electron mass $m^{*}=0.35m_{0}$ are given [31], $m_{0}$ is the electron rest mass and e is the electron charge. Using the aforementioned equations in MATLAB software (2024b-windows/64 bit), when the carrier concentration of ITO varies from 10^19^ to 10^21^ cm^−3^, the corresponding values of $\omega_{p}$ range from 3.02 × 10^14^ rad/s to 3.02 × 10^15^ rad/s. The equation indicates that manipulating *N* will lead to changes in the dielectric constant of ITO. Figure 1a illustrates the relationship between the complex dielectric constant of ITO at a wavelength *λ* = 1550 nm and the free carrier concentration of ITO. By adjusting the electron concentration, the real part of *ε* can approach zero (*N*_ENZ_ = 6.63 × 10^20^ cm^−3^); at this point, the value of $\;\omega_{p}$ is 2.46 × 10^15^ rad/s, and the value of γ is 1.86 × 10^14^ rad/s, implying a transition in the behavior of ITO from a low-loss dielectric (*ε* > 0) to a high-loss metal (*ε* < 0).

## 3. Results and Discussion

Figure 1b presents a comprehensive flowchart of the simulation process. By adjusting the initial doping concentrations of ITO and Si, as well as the thickness of Si, the width of the PN junction depletion region is optimized. Subsequently, the charge density distribution calculated in this step is employed to determine the optical parameters of the waveguide. Ultimately, these parameters are utilized for further high-speed performance analysis.

Figure 1c illustrates the voltage-dependent carrier concentration in the ITO region of the heterojunction device for varying thicknesses of the Si layer. The voltage-dependent carrier concentration curves are obtained by solving the Poisson equation and the drift-diffusion equation simultaneously. This voltage-dependent characteristic is directly related to the material’s own properties, including the electrostatic potential and the free carrier density. The initial doping concentration of the ITO layer in the device is contingent upon the oxygen content during the fabrication process of the ITO film. The carrier concentration decreases with an increase in oxygen content, while the mobility exhibits a trend of initial increase followed by a subsequent decrease as the oxygen content increases [32]. In order to comprehensively consider carrier concentration and mobility to achieve an optimal modulation effect, the initial doping concentration of the ITO layer is set to 7.5 × 10^20^ cm^−3^. Considering the impact of the doping concentration on the depletion layer width and the built-in electric field and potential in a PN junction [33], the initial doping concentration of the Si layer is set to 8.5 × 10^18^ cm^−3^. It is evident that, for a fixed thickness of the Si layer, the carrier concentration decreases significantly with increasing voltage, with a change on the order of magnitude of 10^19^ cm^−3^. Furthermore, as the thickness of the Si layer increases, the dependence of the carrier concentration variation in the ITO layer on voltage also increases, indicating that a larger Si layer thickness is conducive to achieving a greater modulation depth in the modulator. If a high doping concentration is used in Si layer, the waveguide loss will increase; therefore, when the ITO doping concentration is 7.5 × 10^20^ cm^−3^, the Si doping concentration is 8.5 × 10^18^ cm^−3^ with a thickness of 1500 nm, which is more capable of fully leveraging the ENZ effect of ITO. Note that the formulas used here do not take into account other effects that the device may have, such as thermo-optical effects, making the results somewhat limited. The difficulties encountered in the production of this type of heterojunction structure include the precise control of carrier concentration and the contact quality at the heterojunction interface, etc.

**Figure 2:**
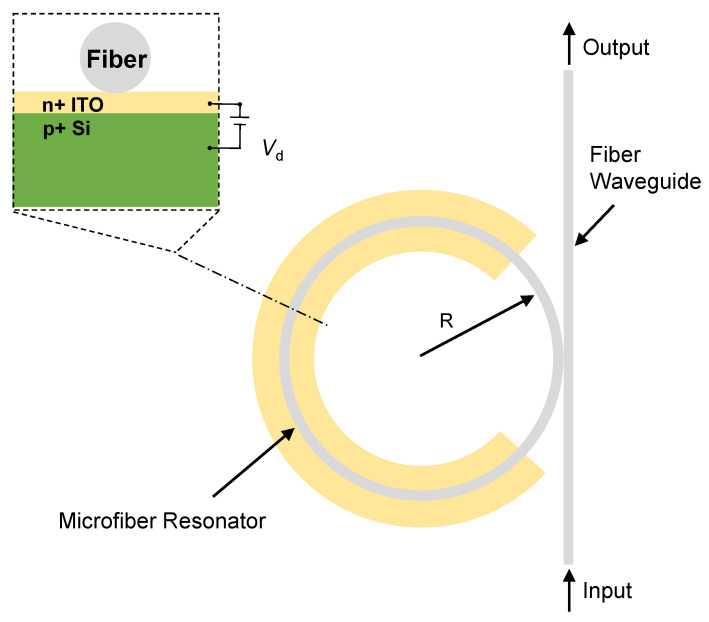
Schematic layout of the ring resonator-based modulator. The inset shows the cross-section of the ring.

Figure 2 presents a schematic diagram of the modulator structure. The device employs a fiber-optic micro-ring resonator (MKR) design for the p-Si/n-ITO heterojunction electro-optic modulator. A 20 nm thick layer of ITO is formed on a 1.5 μm thick Si layer to constitute a carrier depletion structure, and a 2 nm thick, 5 μm radius MKR is transferred onto the ITO surface. The structure of the carrier-depletion type modulator comprises a PN junction that operates in reverse-bias mode. Upon the application of reverse bias, the depletion region expands, within which the carrier density becomes negligible, a condition referred to as the complete depletion approximation; moreover, this device exhibits complete depletion in Si. If the PIN junction structure is adopted, the performance of the modulator is limited by the recombination time of electron–hole pairs after direct voltage is applied, and its modulation rate is not very high, which can only reach several GHz. However, when the reverse voltage is applied, the depletion region in the PIN junction widens and most of it is in the I-layer region, and the region where the carrier concentration changes is very small, so the modulation effect is very small [34]. Soref experimentally demonstrated that carrier depletion affects the absorption coefficient (Δα) and the refractive index (Δ*n*), with the mathematical relationship given by the following equations in the 1550 nm wavelength band [35]:

$$\Delta n=-5.4\times 10^{-22}\Delta N^{1.011}-1.53\times 10^{-18}\Delta P^{0.838}$$

$$\Delta \alpha =8.88\times 10^{-21}\Delta N^{1.167}+5.84\times 10^{-20}\Delta P^{1.109}$$

In these equations, Δ*N* and Δ*P* represent the changes in electron and hole concentrations, respectively, due to carrier depletion. The exponents indicate the power-law dependence of the refractive index and absorption coefficient on the carrier concentrations. These relationships are crucial for understanding the optoelectronic properties of materials under carrier depletion conditions. Note that the formulas used here do not take into account other effects that the device may have, such as thermo-optical effects, making the results somewhat limited.

**Figure 3:**
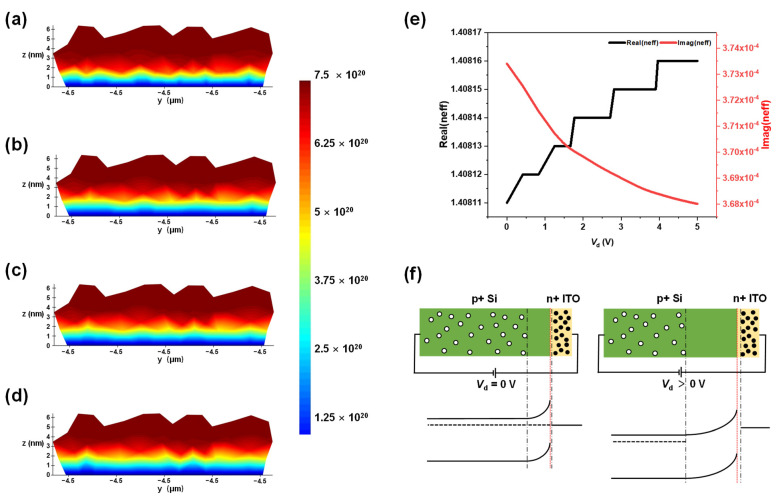
(**a**–**d**) The visualization data of the carrier concentration in the ITO region of the PN heterojunction at 0 V, 2 V, 3 V, and 5 V, respectively. (**e**) The relationship between the real and imaginary parts of the effective refractive index of the optical field in the micro-ring as a function of the externally applied voltage. (**f**) Schematic diagram of the energy band changes in the PN junction before and after the application of reverse bias.

Figure 3a–d display the visualized data of carrier concentration in the ITO region of a PN heterojunction. It is evident that as the voltage increases, the depletion zone within the ITO expands continuously. In the active region where the electron depletion layer induces the ENZ (epsilon-near-zero) effect, the electric field confinement can be enhanced based on the continuity of the normalized displacement field components. This enhancement is quantified by the relationship |*E*_1_| = |*ε*_2_*E*_2_|/|*ε*_1_| [36], where *E*_1_ and *E*_2_ are the normal components of the electric field, and $\varepsilon_{1}$ and $\varepsilon_{2}$ represent the complex permittivities of ITO and SiO_2_ (MKR), respectively. This equation illustrates the intensified electric field confinement accompanying the ENZ effect within the depletion layer. Figure 3e delineates the relationship between the real and imaginary parts of the effective refractive index of the optical field within the micro-ring and the externally applied voltage. It is observable that with the increment of voltage, the real part of the refractive index increases markedly, while the imaginary part exhibits minimal variation. This suggests that the device structure is predominantly designed for phase modulation, which is exceedingly advantageous in applications specific to pure phase modulators. In addition, the real part of ε in the figure shows a stair-step increase with the applied voltage, which is not entirely accurate. The reason for this stepped trend is that the increment in the real part of ε, resulting from minor increases in voltage, is less than 0.00001, which is the limit of the precision in the calculations. Figure S1 (Supplementary Materials) presents the schematic representation of the structure and energy band diagram prior to the contact of the PN heterojunction. Post-contact, a depletion region (space charge region) forms at the PN heterojunction interface. Given that the electron concentration in ITO is significantly higher than in Si, the depletion region and the corresponding energy band bending are primarily localized within the Si layer, as depicted in Figure 3f. Upon the application of a reverse bias, the width of the depletion region is observed to expand.

Figure S2 (Supplementary Materials) illustrates the distribution of the optical field within a fiber-optic micro-ring resonator, with the field intensity biased towards the inner side. By solving the Schrodinger equation of the evanescent wave numerically, the intensity distribution and phase of the evanescent field propagating along the micro-nano fiber ITO optical waveguide are influenced by ITO material [37]. Upon integration with the device, a portion of the evanescent field of the TE mode in the micro-ring couples into the device, interacting with the material and altering the overall optical field distribution. The application of a reverse bias voltage leads to a change in the carrier concentration within the ITO, approaching the Near-Zero Electron Number Density (NENZ), which results in a significant modification of the ITO’s refractive index. The effective refractive index of the micro-ring is significantly altered by the influence of the evanescent field, as demonstrated in equation, where ${neff}_{,i}$ represents the effective refractive index of the MKR.

$$neff\left(v\right)={neff}_{,i}+\int ∆n\left(v\right)dv$$

In turn, this induces a substantial change in the transmission spectrum of the micro-ring resonator T(v), affecting the micro-ring resonance and achieving optical phase modulation.

**Figure 4:**
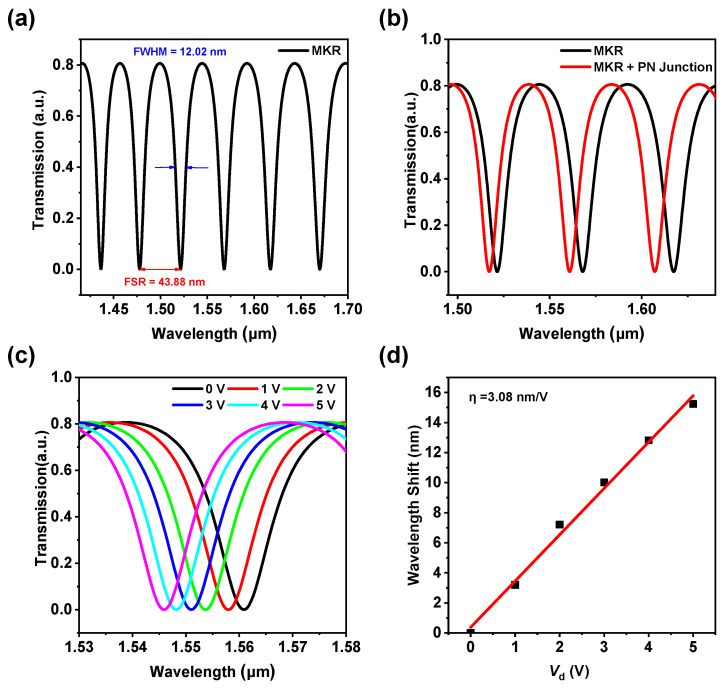
(**a**) Transmission spectrum of the microfiber knot resonator (MKR). (**b**) Transmission spectra of the microfiber knot resonator (MKR) (black solid line) and the MKR with the PN junction (red dashed line). (**c**) Transmission spectra of the all-fiber micro-ring modulator under different reverse bias voltages. (**d**) Scatter plot and fitting curve of the voltage-dependent resonance peak shift.

Figure 4a presents the transmission spectrum of an all-fiber micro-ring resonator. According to the coupled mode theory, the transmission spectrum of the micro-ring resonator (MKR) can be expressed as [38,39].

$$T(v)=(1-\tau )\frac{2k_{\gamma }[1+\sin\left(\beta L\right)]}{1+k_{\gamma }^{2}+2k_{\gamma }\sin(\beta L)}$$

In this formula, τ represents the coupling loss, $k_{\gamma }$ is the coupling coefficient, $\beta =\frac{2\pi neff(v)}{\lambda }$ is the propagation constant, and *L* is the circumference of the MKR. Micro-ring resonators exhibit a resonance phenomenon, which refers to the occurrence of resonance within the micro-ring resonator when the wavelength of the light wave passing through it matches the resonant wavelength ($\lambda_{res}$) of the micro-ring resonator. Specifically, resonance occurs when the optical path length of the micro-ring resonator is an integer multiple of the light wavelength. The resonant wavelength is directly related to the geometric dimensions of the micro-ring resonator and the refractive index of the light wave; any slight change can cause a shift in the resonant wavelength. Therefore, micro-ring resonators are considered an ideal platform for electro-optic modulators. Figure 4a presents the transmission spectrum of an all-fiber micro-ring resonator. It can be observed that the MKR exhibits excellent resonance characteristics, with a free spectral range (FSR) of 43.88 nm, a full width at half maximum (FWHM) of 12.02 nm, and under the condition that the $\lambda_{res}$ is 1550 nm, a quality factor (Q) defined as $Q=\frac{\lambda_{res}}{FWHM}=128.95$. Figure 4b and Figure S3 (Supplementary Materials) depict the transmission spectra of the micro-ring resonator before and after coupling with the PN heterojunction device, as well as the variation curve of the group refractive index of the optical fiber resonator. Based on the resonance characteristics of the micro-ring resonator, the relationship between the spectral shift and the group refractive index can be expressed as [40,41,42]

$$n_{g}=\frac{\lambda^{2}}{L∆\lambda }$$

It is evident that the incorporation of the PN heterojunction device results in an increase in the group refractive index of the optical fiber resonator, consequently leading to a blue shift in the resonance peaks of the micro-ring resonator (MKR). Figure 4c and Figure S4 (Supplementary Materials) present the transmission spectra of the MKR under various voltages applied to the PN heterojunction device. It can be observed that as the voltage increases, the shift in the resonance peak position becomes more pronounced. Figure 4d exhibits the scatter plot and fitting curve of the voltage-dependent resonance peak shift extracted from Figure 4c. It is observable that a significant phase shift can be achieved with very low energy consumption, corresponding to a large modulation depth. Furthermore, the relationship is essentially linear, indicating a high modulation efficiency, reaching 3.08 nm/V.

**Figure 5:**
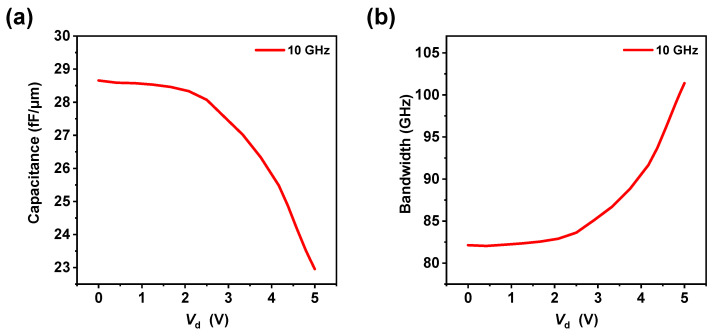
(**a**) Voltage-dependent variation curve of the PN junction capacitance under a 10 GHz small-signal interference. (**b**) Voltage-dependent RC bandwidth limitation curve of the PN junction.

Figure 5a presents the variation curve of the PN heterojunction capacitance under small-signal perturbations with respect to voltage. It is evident that as the voltage increases, the junction capacitance gradually decreases. In a PN junction, there is a depletion region (space charge region) at the interfacial region, which is equivalent to the parallel plate capacitor. When the applied reversed voltage increases, the depletion region will be widened, which is equivalent to the increase in the distance between the two plates of the parallel plate capacitor resulting in a decrease in the junction capacitance. Figure 5b illustrates the cutoff frequency of the PN heterojunction RC constant, according to the formula [43]:

$$f_{Rc}=\frac{1}{2\pi RC}$$

where *R* is the junction resistance and *C* is the junction capacitance. When the drain voltage Vd is increased from 0 V to approximately 1.5 V, the capacitance value remains relatively stable, at around 28.5 fF/μm. As Vd continues to increase beyond 1.5 V, the capacitance value begins to decrease significantly, dropping from about 28.5 fF/μm at 1.5 V to approximately 23 fF/μm at 5 V. When the Vd is increased from 0 V to approximately 2 V, the bandwidth exhibits minimal variation, maintaining a level around 82 GHz. As Vd continues to increase beyond 2 V, the bandwidth experiences a significant increase, rising from approximately 82 GHz at 2 V to exceed 100 GHz at 5 V.

## 4. Conclusions

In this study, we designed and simulated an electro-optic modulator based on an all-fiber micro-ring resonator and a p-Si/n-ITO heterojunction, addressing the limitations of traditional silicon electro-optic modulators in terms of modulation efficiency and bandwidth. The incorporation of ITO, with its high carrier concentration and broad tuning range, significantly enhanced the modulation efficiency while maintaining a large bandwidth. The design of the all-fiber micro-ring resonator eliminated the spatial optical-waveguide coupling process, reducing insertion loss and simplifying the integration procedure. Results indicate that the modulator can achieve significant phase shifts at low voltages, with a modulation efficiency of up to 3.08 nm/V and a bandwidth reaching 82.04 GHz. Our research not only provides a design scheme for an efficient, high-bandwidth electro-optic modulator but also offers new perspectives for the development of future photonic chip technology.

## Data Availability

The original contributions presented in this study are included in the article and Supplementary Material. Further inquiries can be directed to the corresponding author.

## Supplementary Materials

The following supporting information can be downloaded at https://www.mdpi.com/article/10.3390/ma18020307/s1. Figure S1: Schematic Representation of the Structure and Energy Band Diagram Prior to the Formation of a PN Heterojunction; Figure S2: Electric Field Intensity Vector Maps in TE Mode Before and After Incorporating a PN Junction Device; Figure S3: Variation Curve of the Group Refractive Index for an Optical Fiber Resonator; Figure S4: Transmission Spectra of Microknot Resonator (MKR) Under Varying Voltages Applied to the PN Heterojunction Device.
