# Supplementary material for: All-Fiber Micro-Ring Resonator Based p-Si/n-ITO Heterojunction Electro-Optic Modulator
Source: Materials (Basel). 2025 Jan 11;18(2):307. doi: 10.3390/ma18020307 (PMC11766889; doi:10.3390/ma18020307)
Supplement: Supplementary file 1 [file materials-18-00307-s001.zip › materials-3354497-supplementary.pdf]

Article

Supplementary Materials

All-Fiber Micro-Ring Resonator Based p-Si/n-ITO Heterojunction Electro-Optic Modulator

Yihan Zhu[1], Ziqian Wang[1,2], Xing Chen[3], Honghai Zhu[1], Lizhuo Zhou[1], Yujie Zhou[1], Yi Liu[1], Yule Zhang[1], Xilin Tian[1], Shuo Sun[1], Jianqing Li[2], Ke Jiang[4], Han Zhang[1,5], Huide Wang[1,*]

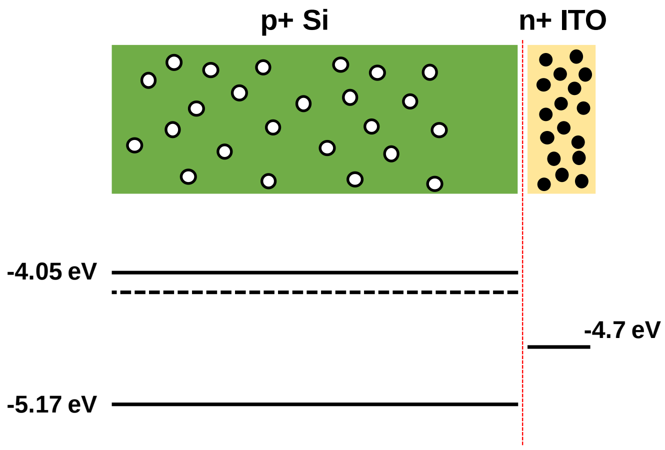

Figure S1. Schematic Representation of the Structure and Energy Band Diagram Prior to the Formation of a PN Heterojunction."

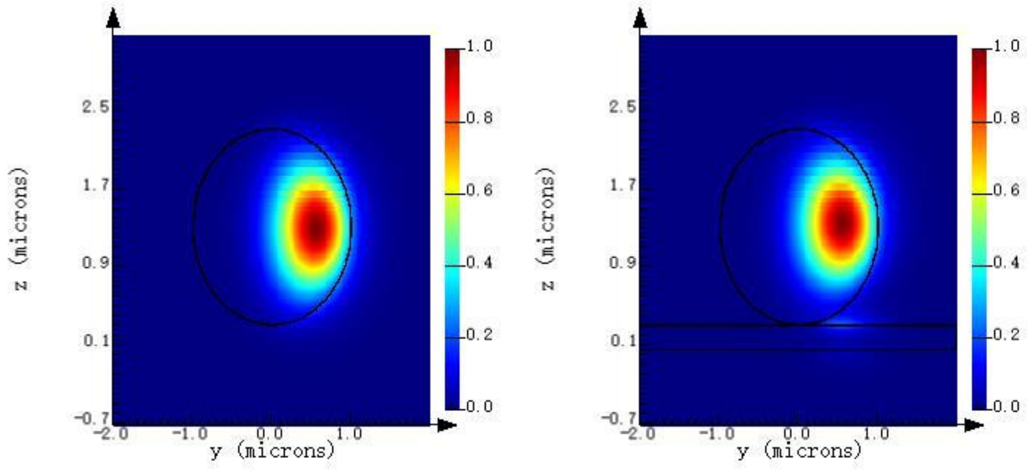

Figure S2. Electric Field Intensity Vector Maps in TE Mode Before and After Incorporating a PN Junction Device

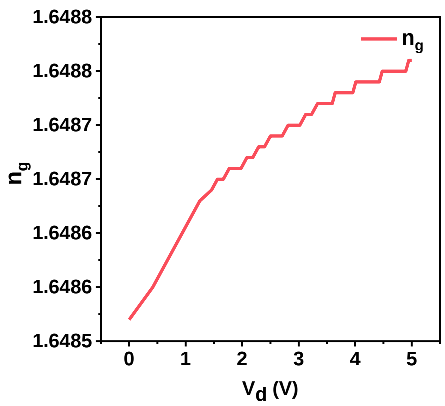

Figure S3. Variation Curve of the Group Refractive Index for an Optical Fiber

Resonator

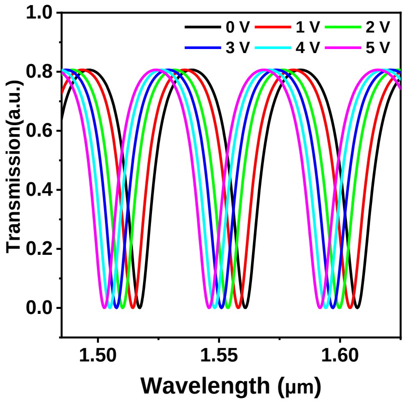

Figure S4. Transmission Spectra of Microknot Resonator (MKR) Under Varying Voltages Applied to the PN Heterojunction Device.
